# Concepts of Neuroinflammation and Their Relationship With Impaired Mitochondrial Functions in Bipolar Disorder

**DOI:** 10.3389/fnbeh.2021.609487

**Published:** 2021-02-26

**Authors:** Luiz Arthur Rangel Cyrino, Daniela Delwing-de Lima, Oliver Matheus Ullmann, Thayná Patachini Maia

**Affiliations:** ^1^Programa de Pós-Graduação em Saúde e Meio Ambiente, Laboratório de Práticas Farmacêuticas of Department of Pharmacy, University of Joinville Region—UNIVILLE, Joinville, Brazil; ^2^Department of Psychology, University of Joinville—UNIVILLE, Joinville, Brazil; ^3^Department of Pharmacy, University of Joinville—UNIVILLE, Joinville, Brazil; ^4^Department of Medicine, University of Joinville—UNIVILLE, Joinville, Brazil

**Keywords:** energy metabolism, mitochondria, bipolar disorder, oxidative stress, neuroinflammation, neuroprogression

## Abstract

Bipolar disorder (BD) is a chronic psychiatric disease, characterized by frequent behavioral episodes of depression and mania, and neurologically by dysregulated neurotransmission, neuroplasticity, growth factor signaling, and metabolism, as well as oxidative stress, and neuronal apoptosis, contributing to chronic neuroinflammation. These abnormalities result from complex interactions between multiple susceptibility genes and environmental factors such as stress. The neurocellular abnormalities of BD can result in gross morphological changes, such as reduced prefrontal and hippocampal volume, and circuit reorganization resulting in cognitive and emotional deficits. The term “neuroprogression” is used to denote the progressive changes from early to late stages, as BD severity and loss of treatment response correlate with the number of past episodes. In addition to circuit and cellular abnormalities, BD is associated with dysfunctional mitochondria, leading to severe metabolic disruption in high energy-demanding neurons and glia. Indeed, mitochondrial dysfunction involving electron transport chain (ETC) disruption is considered the primary cause of chronic oxidative stress in BD. The ensuing damage to membrane lipids, proteins, and DNA further perpetuates oxidative stress and neuroinflammation, creating a perpetuating pathogenic cycle. A deeper understanding of BD pathophysiology and identification of associated biomarkers of neuroinflammation are needed to facilitate early diagnosis and treatment of this debilitating disorder.

## Introduction

Bipolar disorder (BD) is a chronic and recurrent mood disorder characterized by cyclic episodes of depression and mania. Further, some patients may experience psychotic episodes during which there is a high risk of suicide. Depressive and manic episodes are often interspersed with periods of mood stability or euthymia (Goodwin and Jamison, 2007; Hoertel et al., 2013; Sigitova et al., 2017). Global prevalence has been estimated at approximately 1%–2%, but some estimates suggest that it may be as high as 4% (Kessler et al., 2005; Martinowich et al., 2009). BD can be divided into two subtypes, BD I characterized by severe manic and depressive episodes, and the less severe BD II characterized by hypomania and depression. A meta-analysis reported a centrally pooled lifetime prevalence of 1.1% for BD I and 1.2% for BD II (Clemente et al., 2015). Rates vary considerably across studies, however, possibly due to methodological differences. A recent epidemiological meta-analysis of 85 studies, including 67,373 adult patients from 44 countries, found a lifetime BD spectrum prevalence of 1.02%, relatively stable over three decades (Moreira et al., 2017).

The BD concordance rate is significantly higher between monozygotic twins than dizygotic twins, indicating a genetic influence (Barnett and Smoller, 2009). Studies have shown that BD shares pathogenic characteristics with a wide variety of other diseases, including metabolic, cardiovascular, and neurodegenerative diseases (Furman et al., 2019).

These and many other findings reviewed here, suggest that disease etiology is best explained by multiple interactions between environmental factors such as chronic stress and susceptibility genes (Goodwin and Jamison, 2007), altering the brain development, neuroplasticity, chronobiology, neurotransmission, and cell signaling pathways, ultimately leading to neuroinflammation, oxidative stress, and apoptotic cell death (Schloesser et al., 2008; Berk et al., 2011; Szepesi et al., 2018).

Progressive structural and biochemical changes in the prodromal and early stages of the disease, produce a slowly evolving clinical process called neuroprogression. The typical patient exhibits a slow decline in behavioral and cognitive functions associated with a weaker response to treatment (Berk et al., 2011; Borges et al., 2019). This slow progression prevents early diagnosis and rapid initiation of appropriate treatment. Finding an effective treatment regimen usually takes several years, resulting in substantial clinical impairment.

The main objective of this review article is to provide a better understanding of the pathophysiology of BD, especially the contributions of biomarkers such as neurotrophins, cytokines, oxidative stress, metabolic deficiencies, which are directly related to neuroinflammation (Fernandes et al., 2015).

We also seek to understand both the role of neuronal and glial cells, as well as the mitochondrial functions involved in neuroinflammation. These cells have high energy demands in relation to many other cell types, and the mitochondrial dysfunction produces the rupture of the electron transport chain (ETC), which leads to metabolic deficits, oxidative stress, cellular damage, and inflammation. Also, we summarized recent research conducted through biomarkers that were present in blood samples, which assisted in early diagnosis, and treatment response for better BD outcomes. Future research strategies based on these findings, processes, and their impacts on the evolution of the disorder, are discussed in detail below.

## Chronic Stress, Neuroinflammation, and Neuroprogression, as Pathogenic Mechanisms Underlying Bipolar Disorder

A clear relationship has been established between chronic stress and neuropsychiatric pathology, including depression and BD, mediated primarily by dysregulation of the hormonal stress responses (Byrne et al., 2016; McEwen, 2017; Hei et al., 2019). Low levels of glucocorticoid released during acute mild stress can induce a compensatory increase in metabolism and enhance cognitive functions (Miller et al., 2009; Yaribeygi et al., 2017). For instance, glucocorticoid binding to high-affinity receptors can improve working memory and promote long-term memory consolidation by promoting dendritic growth and dendritic spine formation in the hippocampus, amygdala, and prefrontal cortex (Barsegyan et al., 2010; Liston et al., 2013). Under chronic stress, however, glucocorticoid levels decrease as a result of continuous or repetitive long-term stimulation (Hall et al., 2015). This is associated with reductions in glucocorticoid receptor expression and cortisol sensitivity, resulting in hypothalamic-pituitary-adrenal axis adaptation or habituation, particularly during emotional stress which has multiple long-term deleterious effects on neuronal functions, in different areas i.e., hippocampus, anterior cingulate cortex, prefrontal cortex, ventral striatum and insular cortex (Ulrich-Lai and Herman, 2009; Berk et al., 2013; Vieta et al., 2013; Jayasinghe et al., 2015; Rabasa et al., 2015).

Thus, when the challenges imposed by the social and physical environment appear unexpectedly and continuously exceed their limits of intensity and duration. Systems are activated which regulate homeostasis in higher levels of demands, which lead to the concept of allostasis. Thereby, allostasis is the ability to achieve stability by enacting compensatory responses to physiological and environmental stressors. The physiological repetition of allostatic cycles appears to accelerate the disease process (McEwen, 2000; Ganzel et al., 2010), including psychiatric disorders through a mechanism known as allostatic load (Rios, 2014). The allostatic load hypothesis was developed to explain the substantial clinical changes observed in the pathologies, and how these cumulative changes are reflected by the progression of the disease, leaving the cells and organs inefficient (i.e., pathogenic; McEwen, 2000; Grande et al., 2012). This concept has been transferred to brain diseases and has been referred to as neuroprogression (Berk et al., 2009, 2011; Salagre et al., 2018). Particularly in BD patients, the repeated episodes of depression and mania over decades, enhance the vulnerability to stress, further reducing the patients’ recovery capacity and accelerating disease process, impairing several functions like the reduction of neural plasticity, consequently reducing the memory capacity, irregular emotional responses, mood control, and decision making (Jansen et al., 2013; Vasconcelos-Moreno et al., 2017; Lacroix, 2019). This process can be better understood through the effects in the brain cells and their mitochondria, which have pro-inflammatory peripheral cytokine receptors, such as interleukins (IL)-6, IL-10, and tumor necrosis factor α (TNF-α), which respond by releasing second messengers, stimulating the production of more cytokines by the CNS. The initial stimulation may derive from damaged tissues releasing cytokines into the bloodstream, or by inflammatory stimulation of peripheral afferent neurons (Irwin and Cole, 2011; Fregnan et al., 2012; Zuccoli et al., 2017). Upon arrival in the brain, these proteins activate other cells and biochemical reactions, which enhance the allostatic load and can be modulated by multiple mechanisms like leukocytosis, a reduction in lymphocytes and natural killer cells, increased CD4+/CD8+ ratios, more proinflammatory cytokines (IL-1, IL-6, and TNF-α), cytokine receptor expression, and activation of the downstream nuclear factor kappa B (NF-κB) stress response pathway (Byrne et al., 2016; Gulati et al., 2016). At the cellular level, several of these effects are associated with altered calcium signaling. In chronic levels of cortisol, the expression of L-type calcium channels is upregulated by glucocorticoids promoting a greater Ca^2+^ influx into the cells promoting protease and phospholipase activation (Joëls et al., 2012, 2013; Merkulov et al., 2017). Also, when an overloaded calcium entry occurs, it leads to the opening of the mitochondrial permeability transition pore, and outer mitochondrial membrane permeabilization, respectively, facilitating the release of cytochrome c through the mitochondrial outer membrane, which triggers the caspase-3-dependent apoptosis cascade (Pereira et al., 2012; Perier et al., 2012; Di Meo et al., 2016). Furthermore, this increased calcium entry reduces ETC-coupled proton export, resulting in a reduced adenosine triphosphate (ATP) synthesis (Lin et al., 2012; Zhao et al., 2019). Furthermore, the extrinsic apoptosis pathway is triggered by the ligation of TNF-family death receptors at the cell surface. Receptor ligation can result in the recruitment of the Fas-associated death domain protein, which in turn binds procaspase-8 molecules, allowing autoproteolytic processing and activation of caspase-8, the principal effector of the extrinsic apoptosis pathway (Youle and Strasser, 2008; Machado-Vieira et al., 2009; Mitochondrial dysfunctions can also trigger excessive production of free radicals, leading to oxidative stress that eventually reduces metabolism and induces neuroplastic dysfunction, contributing to apoptosis by altering the structure of lipids, proteins, and DNA molecules (Machado-Vieira et al., 2007; Vakifahmetoglu-Norberg et al., 2017).

All of these elements are involved in the chronic stress response, which demonstrates their close relationship with the immune system, where all of them causes a decrease in the capacity for neuronal repair, and mitochondrial transport to synaptic regions *via* the cytoskeleton with further neuronal dysfunction and death (Mizisin and Weerasuriya, 2011; Lacroix, 2019). There is compelling evidence that BD arises through alterations in the synapses and critical circuit functions, rather than an imbalance of specific neurotransmitters mediating affective and cognitive functions (Martinowich et al., 2009; Scaini et al., 2020). Furthermore, prolonged metabolic dysregulation, deficient neurotrophin (NT) signaling, oxidative stress, and neuroinflammation, which may contribute to the increased frequency and severity of manic and depressive episodes, as well as other sequelae with age (Heneka et al., 2010; Kim et al., 2020). These neurological and behavioral abnormalities, in turn, will interfere with the patient’s personal and professional life, leading to further stress-related pathogenesis. BD patients also exhibit an increase in psychiatric and medical comorbidities, which also may be associated with an imbalance of these mediators (Kapczinski and Streb, 2014; Rowland et al., 2018).

Increased levels of proinflammatory cytokines in the CNS stimulate the activation of immune cells, including macrophages, monocytes, and microglia. Rising inflammatory cytokines in the CNS appear to depend on the activation of microglia (McEwen, 2017).

The role of microglia and the participation of proinflammatory mediators in neuroinflammation will be discussed from this point forward. The CNS hosts a heterogeneous population of resident myeloid-derived immune cells that regulate communication between the nervous, vascular, and immune systems. Most prominent among these are the parenchymal microglia, which account for up to 16% of the total cell number in some areas of the human brain (Norden and Godbout, 2013). Microglia perform essential homeostatic functions under non-pathological conditions, including regulation of neural circuit development (Squarzoni et al., 2014) through the release of neurotrophins such as brain-derived neurotrophic factor (BDNF; Parkhurst et al., 2013), clearance of apoptotic cells and cellular debris, and synaptic pruning (Paolicelli et al., 2011). Microglia are also critical regulators of neuroinflammation in response to brain trauma and various pathogenic insults (Gomez-Nicola et al., 2014). Until recently, only circulating monocytes were thought to replenish tissue macrophage populations, including CNS microglia. However, new research suggests the presence of two ontogenetically and genetically distinct myeloid populations of microglia and nonparenchymal macrophages in the meninges, perivascular spaces, and choroid plexus (Jakubzick et al., 2013; Davies and Taylor, 2015; Herz et al., 2017). In rodents, microglial progenitors derived from the yolk sack appear on an embryonic day (E) 8.5 (Ginhoux et al., 2010; Gomez Perdiguero et al., 2015) distributed in the brain before birth, and remain a stable population throughout life. In contrast, other CNS macrophages likely originate from monocytes derived from the bone marrow (Ajami et al., 2011; Kierdorf et al., 2013), are short-lived after birth and show rapid turnover through proliferation and apoptosis (Aguzzi et al., 2013; Prinz and Priller, 2014), which renews the entire population several times over a lifetime (Askew et al., 2017). Rodent cell transplantation experiments (Hickey et al., 1992) and observations following bone marrow transplantation (Yang et al., 2013; Barr et al., 2015) also indicate that some monocytes in the blood and perivascular macrophages can infiltrate into the CNS parenchyma (Mildner et al., 2007; Kierdorf et al., 2015). Despite their distinct origins, CNS microglia and macrophages are morphologically similar and share certain functions. However, microglia cannot be replaced by monocyte-derived macrophages due to their specific gene expression patterns and unique functions (Goldmann et al., 2013; Ginhoux et al., 2016; Prinz and Priller, 2017). In addition to microglial cells, nonparenchymal and endothelial cells regulate neural-immune function by maintaining the BBB, promoting angiogenesis, regulating the composition of the cerebrospinal fluid, and controlling vascular tone (He et al., 2016). Various dendritic cells, mast cells, monocytes, and granulocytes complete the CNS immune system (Kierdorf et al., 2015; Goldmann et al., 2016). Mast cells are one of the few cells that migrate to the CNS under both physiological and pathological conditions, where they reside in the neuronal parenchyma (Sayed et al., 2010) and function as pathogen sensors and modulate inflammation by recruiting other immune cells to specific target regions (Skaper et al., 2013). Many of these cells acquire anti-inflammatory or pro-inflammatory phenotypes (Brendecke and Prinz, 2015), and it is the balance between these phenotypes that determines overall neuroinflammatory status and the progression of neuroinflammatory diseases (Goldmann et al., 2016). Even in their inactivated resting state, microglia continually search for signs of potential threats to the CNS (Hellwig et al., 2013). Multiple pathways are activated by chemical signals from infection, trauma, endogenous and exogenous toxins, and the loss of constitutive anti-inflammatory signals. Studies have shown that microglia and brain macrophages can differentiate into two distinct phenotypic groups, the classically activated (M1) and alternatively activated (M2) populations (Chawla, 2010; Geissmann et al., 2010). These reactive phenotypes have distinct protein and non-coding mRNA expression profiles, release unique cytokines and chemokines, and have different phagocytic activities. The reactive behavior of M1 microglia can eliminate the initial activation trigger (such as a pathogen) with or without the support of other resident or invasive immune cells. This loss of the pathogenic stimulus leads to a more repair-oriented microglial profile and eventual reversion to the initial resting state (Arcuri et al., 2017; Tohidpour et al., 2017). Thus, the microglia produce an immune response during inflammatory conditions, moderating potential damage to the CNS and aiding in tissue repair and remodeling (Kingwell, 2012; Hellwig et al., 2013). Furthermore, in the early stages of diseases, symptoms may be followed by microglial polarization to M1 (Duffy et al., 2010; Yutaka and Kenji, 2014; Ginhoux and Guilliams, 2016). This M1 phenotype can produce proinflammatory cytokines and oxidative metabolites that cause additional damage, such as TNF-α and IL-6 and IL-1 (Colton, 2009; Miller and Raison, 2016). Activation of the M2 phenotype by IL-4, IL-13, or IL-10 (Nguyen et al., 2011; Nakagawa and Chiba, 2014) negatively regulates M1 function, thereby suppressing inflammation and promoting tissue repair and wound healing, consequently attenuating symptoms and restoring tissue homeostasis (Kawabori and Yenari, 2014). In certain chronic conditions, however, some cells may not return to a complete resting state, whereas others remain post-activated microglia. If M2 microglia polarization is insufficient, M1 microglial functions are maintained and induce sustained inflammation and progressive neural network dysfunction. In turn, symptom severity may gradually increase according to the frequency of M1 polarization (Yutaka and Kenji, 2014; Bachiller et al., 2018). These cells may maintain subtle changes, such as transcriptional activity, that modulate their sensitivity to anti-inflammatory signals or alter responses to subsequent stimulation. Sustained M1 activity may even lead to neuronal degeneration (Arcuri et al., 2017). A recent study reported that there was an M1 dominance in one of three BD patients during the manic state, and a downregulation of M2 markers during the manic state in all three patients, suggesting that the M1/M2 balance may indeed contribute to BD symptoms. The researchers showed that the gene profiling patterns are different between manic and depressive states (Ohgidani et al., 2017).

However, while activated microglia have demonstrated neurotoxic effects, responses may be very different *in vivo* compared to the commonly used *in vitro* models (Hellwig et al., 2013) due to the absence of inhibitory factors such as CD200, CX3CL1, CD22, and CD172, which maintain microglia attenuation *in vivo* (Ransohoff and Cardona, 2010; Prinz et al., 2011). Blocking even one of these inhibitory factors results in profound changes in microglial reactions, often causing a disproportionate immune response and occasionally cytotoxic responses (Hoek et al., 2000; Cardona et al., 2006). With all that, neurons may be damaged or functionally impaired when microglial activation is dysregulated, and microglia-mediated inflammation is intense, as observed in chronic brain pathologies. This inflammatory neuronal damage can contribute to the progression of neurological disease, and possibly psychiatric diseases such as BD (Perry et al., 2010; Kettenmann et al., 2011; von Bernhardi et al., 2015).

## Energy Deficits in Neuronal Mitochondria and Its Possible Relationship to Psychiatric Disorders

Neurons contain large numbers of mitochondria to supply the energy required for the maintenance of ion gradients and electrical signaling, neurite growth, long-distance axonal transport (mitochondria to distal synapses), calcium homeostasis, and calcium signaling. Neurons are highly energy-demanding cells. A single cortical neuron at rest consumes approximately 4.7 million ATP molecules per second to execute various biological functions, including the maintenance of ionic gradients critical for electrophysiological signaling. In the human brain, the ATP utilization rate is three times higher in gray matter than white matter (Zhu et al., 2012, 2018), and gray matter neurons are responsible for approximately 20%–25% of all systemic oxygen and glucose consumption (Attwell and Laughlin, 2001). Proper mitochondrial functions are, therefore, critical for neural purposes.

Although the entire neuron requires energy, some sites display higher energy demand, including presynaptic and postsynaptic terminals that mediate neurotransmission, active growth cones or axonal branches, which regulate short- and long-term plasticity, and also Ranvier’s nodes, where the transmembrane ion flux is the highest (Zhang et al., 2010; Sheng and Cai, 2012). This is only possible because mitochondria are highly dynamic, and the relevance of these dynamic processes to BD and other psychiatric disorders is related to the constant changes in the mitochondrial number. It produces an altered mitochondrial distribution and a defective transport. The rapid movement of axonal mitochondria is a primary mechanism, underlying spontaneous and neural activity-dependent synaptic remodeling, being altered under certain conditions, such as stress and axonal trauma (Cataldo et al., 2010; Sheng and Cai, 2012; Sun et al., 2013; Chu, 2019). Sustained local mitochondrial energy production in the presynaptic region is critical for synaptic vesicle release. For instance, a drop in ATP levels in hippocampal synaptosomes reduces synaptic vesicle release and alters the cytosolic calcium concentration (Ivannikov et al., 2013). The postsynaptic site also has extensive energy requirements. A combined proteomics and mass spectroscopy study by Föcking et al. (2016) found high cytoskeletal and signaling protein densities in the postsynaptic region, facilitating the movement of receptors and activating complexes critical for standard synaptic transmission and plasticity. Aberrant synaptic plasticity is implicated in neuropsychiatric disorders such as schizophrenia and BD and may stem from mitochondrial dysfunction and reduced metabolism (Akula et al., 2015; Forero et al., 2016). As shown in Table 1, the mitochondrial protein synthesis combined involves a total of 37 nuclear DNA (nDNA), and mitochondrial DNA (mtDNA) genes. Many of these proteins are involved in both oxidative phosphorylation (OXPHOS) and ETC, and any change in genes can drastically interfere with metabolism (Björkholm et al., 2015; Garcia et al., 2017; Kang et al., 2018). Thus, multiple lines of evidence implicate mitochondrial dysfunctions in BD, including the ~20-fold higher incidences of BD symptoms in patients with mitochondrial diseases. The research reported the identification of mitochondrial DNA deletions, polymorphisms in some BD cases, aberrant up- or downregulation of various mitochondrial genes, BD-like behavioral phenotypes in mouse models with mitochondrial gene mutations. Furthermore, there were differences in mitochondrial morphology, distribution, and metabolite levels between BD patients and controls (Kato, 2017).

**Table 1:** Structural codifications of Oxidative Phosphorylation System (OXPHOS) complexes from nDNA and mtDNA.

Structural codification of OXPHOS complexes					
Complex	I	II	III	IV	V
nDNA encoded polypeptides	≅ 38	4	10	10	16
mtDNA encoded polypeptides	7	0	1	3	2

**Figure 1:**
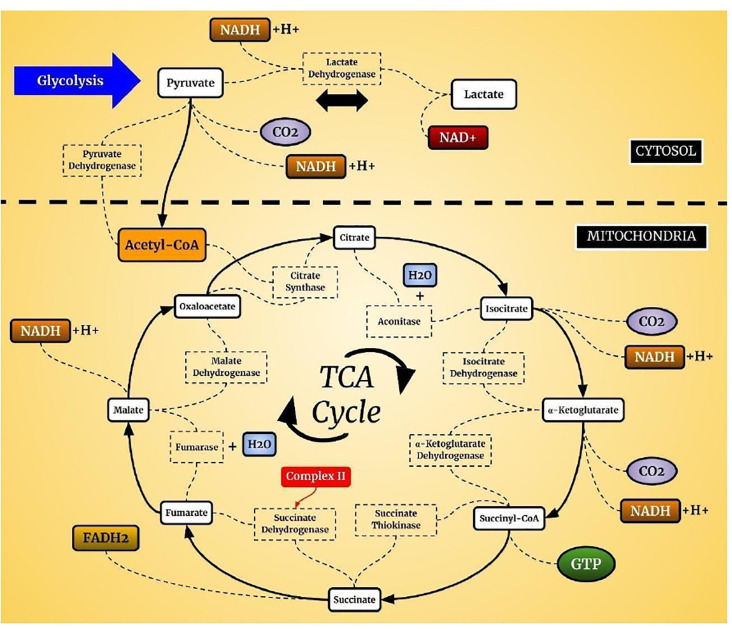
Tricarboxylic acid cycle (TCA) cycle. In aerobic organisms, glucose is oxidized to CO_2_ and H_2_O. The pyruvate present in the cell cytosol is oxidized to acetyl-CoA which can enter the TCA cycle. This cycle is composed of a complex of enzymes located in the mitochondrial cytosol of eukaryotic cells.

**Figure 2:**
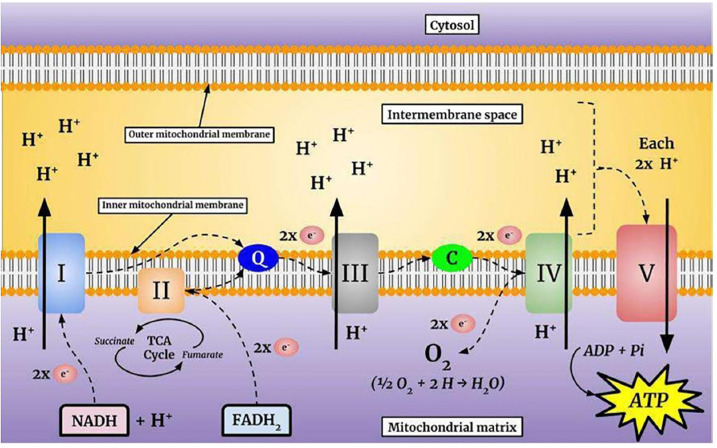
The mitochondrial electron transport chain (ETC). The flow of electrons through the complexes is energetically coupled to the pumping of protons into the intermembrane space. This process produces an electrochemical gradient that stores the energy necessary for adenosine triphosphate (ATP) synthesis.

Mitochondria produce ATP from metabolites through two continuous biochemical processes, the tricarboxylic acid cycle (TCA) and OXPHOS (Vidyasagar, 2015). ATP generation within the mitochondrial matrix requires interconnected processes (Cooper and Hausman, 2006; Kühlbrandt, 2015). First, fatty acids and the glycolytic degradation product pyruvate are converted into acetyl-CoA *via* matrix enzymes (Cronan and Laporte, 2005) and enter the TCA cycle (Figure 1), which generates electron-rich NADH and FADH_2_ as sources for ETC complexes I and II, respectively (Enríquez, 2016). The electrons are transferred to Coenzyme Q10, which is essential due to its antioxidative properties (Bentinger et al., 2010). Coenzyme Q10 transfers the electrons to Complex III, where they are transported to Complex IV by cytochrome c (Alvarez-Paggi et al., 2017). In Complex IV, electrons are transferred to molecular oxygen to form water (Enríquez, 2016; Milenkovic et al., 2017). Finally, hydrogen is pumped through Complex V to store energy for ATP formation from ADP and inorganic phosphate, which is coupled to controlled re-entry of protons in the mitochondrial matrix (Figure 2; Walker, 2013; Angrimani et al., 2015).

The interest of neuropsychiatry in the TCA cycle and oxidative stress focused on the studies related to the production of brain energy. The TCA cycle plays an important role since it is responsible for the reactions that generate the substrates for OXPHOS that occur in ETC. The expression of levels or activities of several TCA enzymes are altered in the brains of BD patients, which may contribute to both neuronal energy deficits and oxidative stress (Blass and Brown, 2000; Zuccoli et al., 2017). Research with bipolar patients and animal models observed a reduction in TCA cycle enzymes (Lee et al., 2007; Valvassori et al., 2013). In oxidative stress, it is essential to note that mitochondria are the primary source of free radicals, and are generated mainly through the ETC, during the energy production from glucose and oxygen, it generates oxidative stress (Barbosa et al., 2010; Mandavilli et al., 2018). Initially, we will describe the main processes, functions, and losses in oxidative stress, and later, the changes that occur in BD, which are related to the section on biomarkers.

**Figure 3:**
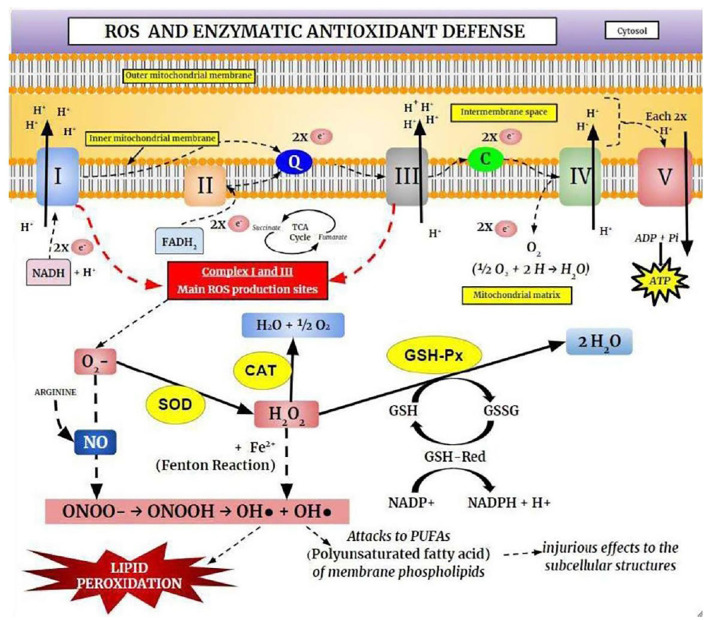
The Oxidative Phosphorylation System (OXPHOS) and the enzymatic antioxidant defense. Electrons derived from cellular metabolism reach Complexes I or II through NADH or FADH_2_, respectively. These electrons are then transferred to Coenzyme Q10 (ubiquinone), which carries electrons from Complexes I or II to Complex III. In Complex III, electrons are displaced from cytochrome b to cytochrome c and consequently transferred to Complex IV (cytochrome c oxidase), where they reduce O_2_. These electrons are transported through mitochondrial protein complexes and are coupled to proton pumping into the intermembrane space. Complex V uses the generated electrochemical gradient for ATP synthesis. Complexes I and II are responsible for the production of superoxide anion (O_2_−), which is removed by the antioxidant enzyme superoxide dismutase (SOD). This process produces hydrogen peroxide (H_2_O_2_), which is removed by catalase (CAT) and glutathione peroxidase (GSH-Px) enzymes. Superoxide and H_2_O_2_ can be converted into highly reactive hydroxyl radicals (OH·), causing lipoperoxidation and cellular injury.

Free radicals are molecules that have at least one unpaired valence electron, resulting in chemical instability and high reactivity with other molecules being continuously produced under physiological conditions (Figure 3). However, the free radicals, both the reactive oxygen species (ROS) and reactive nitrogen species (RNS), are derived from both endogenous sources (mitochondria, peroxisomes, et cetera), and exogenous sources (alcohol, heavy metals, et cetera; Phaniendra et al., 2015).

The physiologically and pathologically relevant free radicals include; superoxide (O_2_•−), hydroxyl (OH•), nitric oxide (NO•), peroxyl-lipids (LOO−), and hydrogen peroxide (H_2_O_2_), and they are formed *via* enzymatic and non-enzymatic reactions (Reynolds et al., 2007; Collin, 2019). Superoxide is the leading free radical species formed by the ETC, and thus, production is enhanced in metabolically active neurons. The OH• is the most reactive biological species and can damage for instance ETC complexes I, II, and III. It can also lead to Fe-S electron center complex malfunction in the TCA cycle and affect mtDNA, leading to further oxidative stress resulting in a reduction in mitochondrial energy production (Federico et al., 2012; Ghezzi and Zeviani, 2012; Voets et al., 2012; Kausar et al., 2018). In contrast, hydrogen peroxide is not strongly reactive but can be toxic due to its long half-life, damaging the membrane permeability (Barreiros et al., 2006).

In healthy cells and tissues, multiple enzymatic and non-enzymatic antioxidant defense systems can reduce the damage caused by free radical production. However, most ROS are neutralized by endogenous enzymatic antioxidants which consist of diverse proteins that metabolize free radicals, reduce oxidized molecules, and peroxidized lipids, being the best known, the superoxide dismutase (SOD), catalase (CAT), GSH S-transferase, y-glutamylcysteine synthetase, GSH peroxidase (GSH-Px), and GSH reductase (Jeeva et al., 2015; Kurutas, 2016). When free radical production is greater than the endogenous antioxidant capacity, it leads to oxidative stress (Barreiros et al., 2006; Barbosa et al., 2010). Among the most pathogenic results of oxidative stress in neurons, is self-perpetuating membrane lipid peroxidation, which results in reduced membrane fluidity and barrier function (Ademowo et al., 2017). SOD acts as the primary protective enzyme against oxidative stress and DNA damage in mitochondria by catalyzing the dismutation of O_2_•− into H_2_O_2_ and O_2_ (Gill and Tuteja, 2010; Krishnamurthy and Wadhwani, 2012). In turn, H_2_O_2_ is converted to H_2_O and O_2_ in most tissues by CAT (Barbosa et al., 2010). At low concentrations, H_2_O_2_ regulates several physiological processes, however, at higher concentrations, it damages cells by reacting with cellular iron to form hydroxyl radicals. Therefore, CAT is critical for limiting H_2_O_2_-induced damage (Ighodaro and Akinloye, 2018). GSH-Px inhibits lipid peroxidation, thereby preventing loss of membrane function. Like CAT, it acts by catalyzing the reduction of H_2_O_2_ or RHO_2_ to H_2_O by GSH, which is concomitantly oxidized to form the disulfide-bonded dimer GSSG (Espinosa-Diez et al., 2015).

It is important to highlight that the brain is particularly vulnerable to oxidative damage because it uses a high oxygen utilization rate, associated with weak defense of antioxidants, and a constitution rich in lipids, favoring the oxidative damage in neuronal cells (Salim, 2017). Thus, alterations in antioxidants and various oxidation products suggest a possible link between oxidative stress and BD. Evidence demonstrates that ROS act as essential second messengers in innate and adaptive immunity (West et al., 2011; Kamiński et al., 2013), stimulating proinflammatory cytokine generation (including IL-1B, IL-8, TNF-α, and interferons) during the immune response to control pathogens and repair tissue damage (Chen and Nuñez, 2010; Mittal et al., 2013). Therefore, in BD, a probable hypothesis is that a higher oxidative stress load is generated by a fundamental disturbance in mitochondrial functions, aggravating the disease. Based on these findings, improved mitochondrial functions is a potentially promising strategy for BD treatment.

## Biomarkers Related to Neuroinflammation in Bipolar Disorder

Collectively, the findings mentioned above indicate that BD should be treated as a multisystem inflammatory disease. An analysis of biomarkers for inflammation and oxidative stress showed that patients with acute BD onset have a significantly higher systemic toxicity than healthy controls, although not as severe as sepsis (Pfaffenseller et al., 2013). Although consequent allostatic overload associated with neuroinflammation in BD patients has been present, a causal link between systemic toxicity and biomarkers has not yet been established. As such, there is an increasing interest in identifying peripheral biomarkers that could function as indicators for systemic and cellular toxicity in BD (Kapczinski et al., 2011; Frank et al., 2014). Specific biomarkers or combinations may be associated with the degree of disease activity during active periods or remission. Notably, some systemic markers have already been implicated as mediators of BD allostasis, and in neuroinflammation (Juster et al., 2013). Thus, such studies have improved our understanding of the disease’s activity and progression, providing clues to new novel therapeutic targets.

While there is still no reliable set of biomarkers for early diagnosis, many show promise for disease detection and treatment evaluation. These biomarkers fall into three categories: (1) imaging signs; (2) genetic loci; and (3) metabolic molecules. Category 3, includes various substances that are derived from neuronal and glial cells, such as the neurotrophins BDNF, glia-derived neurotrophic factor (GDNF), and neuronal growth factor. The pro-inflammatory cytokines IL-6, IL-1, and TNF-α, TCA markers such as citrate synthase, succinate dehydrogenase, and malate dehydrogenase, and oxidative stress-related markers including SOD, CAT, GLUT-Px, 3-nitrotyrosine, and products of lipid peroxidation (Thiobarbituric Acid-Reactive Substance; de Sousa et al., 2015; Scaini et al., 2016). In this review article, we will focus mainly on the last group (metabolic molecules), associating the alterations found in BD and their relationship with neuroinflammation.

### Neurotrophins

Neurotrophins are small secreted proteins that promote multiple neuronal responses through surface receptor binding and activation of several downstream kinase signaling pathways. More than 50 neuronal growth factors are expressed in the mammalian brain. The NT family members BDNF, GDNF, NGF, NT-3, and NT-4/5 increase cell survival by stimulating axonal regeneration following injury and inhibiting apoptotic protein cascades, and promote multiple forms of neurite and synaptic plasticity (Machado-Vieira et al., 2009; Pereira et al., 2012). BDNF activates two distinct receptors, the NT p75 receptor, and the Trk tyrosine kinase receptor. These two receptors can have opposing actions depending on ligand availability and cellular context (Mocchetti and Brown, 2008; Sasi et al., 2017). Both receptors regulate development, survival, repair, cortical dendritic growth, and plasticity as observed, for example, in the visual cortex and its connections (Huberman and McAllister, 2002; Sutton and Schuman, 2006). The p75 receptor signals mainly through stress-associated pathways such as JNK, p53, and NF-κB, while Trk receptors activate the Akt and mitogen-activated protein kinase/extracellular regulated kinase (MAPK/ERK) pathways. Activation of the Trk receptor by BDNF phosphorylates target proteins such as phospholipase C, phosphatidylinositol-3 kinase (PI3K), and ERK1/2 (Kaplan and Miller, 2000; Park and Poo, 2013). The MAPK/ERK pathway initiates a cascade that inhibits pro-apoptotic proteins and increases the expression and phosphorylation (activation) of the transcription factor nuclear cAMP response element (CREB), which upregulates the expression of neurotrophic/neuroprotective proteins such as Bcl-2 and BDNF (Machado-Vieira et al., 2009; Benito and Barco, 2010). Chronic cell stress can result in the dysregulation of any component of the BDNF–MAPK/ERK–CREB pathway. For example, overstimulation by cortisol can negatively regulate CREB phosphorylation and subsequently decrease the transcription of NT genes such as BDNF (Kandel, 2001; Carlezon et al., 2005). This fact is especially vital in psychiatric illnesses and may contribute to the pathophysiology of BD (Berk et al., 2009; Tramontina et al., 2009).

Out of these, BDNF is the most widely distributed and is also the most studied in BD. The initial meta-analyses have reported reduced serum BDNF in BD patients during manic or depressive states, compared to euthymia. These discoveries have been found both in the serum of living BD patients, as well as in postmortem neurons (Knable et al., 2004; Sen et al., 2008; Fernandes et al., 2009; Lin, 2009). However, other meta-analyses and longitudinal studies have demonstrated that the reduced BDNF levels associated during BD manic and depressive phases were responsive at clinically useful drugs like lithium can elevate BDNF expression in the brain (Lang et al., 2007; Yang et al., 2009; Schmidt et al., 2011). More recently, Fernandes et al. (2015) performed a systematic review and meta-analysis evaluated serum and plasma BDNF levels in BD including a total of 52 studies with 6,481 participants, showing that, compared to healthy controls, peripheral BDNF levels are reduced to the same extent in manic and depressive episodes, while BDNF levels are not significantly altered in euthymia. The researchers showed the BDNF levels were negatively correlated with the severity of both manic and depressive symptoms. However, they found no evidence for a significant impact of illness duration on BDNF levels. Also, in plasma peripheral, BDNF levels increase after the successful treatment of an acute manic episode, but not of a depressive one demonstrating that BDNF is a potential biomarker of disease activity in BD, but not a biomarker of the stage (Panaccione et al., 2015; Roda et al., 2015). While it is not always clear whether reduced BDNF is a cause or consequence of BD-related pathology, there is a suggestive association between changes in brain BDNF levels and BD. The reduced serum BDNF in the brains of BD patients exhibits a variety of gross and fine morphological changes that become more pronounced with repeated episodes and disease duration (Tramontina et al., 2009; Olsen et al., 2013). For instance, reductions in the density of oligodendrocytes and myelination in BD patients showed brain white matter abnormalities have been observed in subgenual prefrontal cortex layer VI, caudate nucleus, and the hippocampus along with signs of necrosis and apoptosis (Mechawar and Savitz, 2016; Ganzola and Duchesne, 2017). Also, reduced neuronal somal size, increased somal density, and reduced dendritic spine density have been observed in the anterior cingulate cortex of BD patients. These changes may explain the associated impairments in cognition and judgment (Vostrikov et al., 2007; Konopaske et al., 2014).

As reported above, both cross-sectional and longitudinal studies have indicated that administration of antidepressants and mood stabilizers such as lithium and valproate has normalized BDNF, promoting so, a neuroprotection stress-induced, protecting the cells through anti-apoptotic pathways activation (Colucci-D’Amato et al., 2020). It can also increase the gray matter promoting neurogenesis in the subventricular zone of the lateral ventricle and subgranular zone of the hippocampal dentate gyrus, and improve cognitive functions such as learning and memory, both in animal and human studies (Sassi et al., 2002; Gould, 2007; Kempton et al., 2008; Machado-Vieira et al., 2009; Hashimoto, 2010; Corena-McLeod et al., 2013; Yu and Greenberg, 2016). However, the utility of BDNF as a biomarker during the early or late phases of the disease remains to be determined in longitudinal studies. Further studies are also needed to identify whether BDNF modulation can reduce acute episodes, and promote the possibility for patients to return to euthymia.

Other neurotrophins and neurotrophic factors are also altered in BD patients, reinforcing the hypothesis that impairments in neuroplasticity are involved in pathophysiology (Scola and Andreazza, 2015). Both NT-3 (Fernandes et al., 2010) and NT-4/5 (Walz et al., 2009) were increased during manic and depressive episodes compared to euthymic patients or healthy controls. However, studies on GDNF changes are conflicting. Barbosa et al. (2011b) found increased plasma GDNF levels in euthymic patients compared to manic patients and controls, while Rosa et al. (2006) observed increased GDNF levels in manic and depressive patients, but not in euthymic patients, compared to controls. In another study, GDNF serum levels were reduced in patients during manic and depressive episodes but increased after mood stabilizer treatment (Zhang et al., 2010). Additional studies are needed to assess whether peripheral GDNF levels correlate with CNS levels.

### Inflammatory Cytokines

Both peripheral immune cells and resident brain cells, such as astrocytes, oligodendrocytes, and microglial cells, are associated with elevated pro-and anti-inflammatory cytokines (Barbosa et al., 2011a). The imbalance between them, have been implicated in neuroinflammation, causing toxicity and apoptosis of neurons and glial cells (Dong and Zhen, 2015; Réus et al., 2015; Muneer, 2016), which is associated with neuroprogression in BD, as well as other psychiatric diseases (Kato et al., 2013).

The prevailing hypothesis is that the immune system is chronically activated in BD mainly through microglial activation, which leads to an imbalance of pro-and anti-inflammatory cytokines and chemokines, which in turn can deregulate moods. This hypothesis arose after a significant number of patients with hepatitis C (treated using IFN-α), experienced depressive or manic symptoms (Hoyo-Becerra et al., 2014). It is not clear how peripheral cytokines affect inflammatory processes in the CNS since they do not readily cross the BBB under physiological conditions. Furthermore, postmortem studies have shown an increase or decrease in various pro- and anti-inflammatory factors in the prefrontal cortex, hippocampus, and cingulate gyrus of BD patients (Rao et al., 2010; Sneeboer et al., 2019). Also, many of the changes in cytokine levels found among bipolar patients, are similar to those observed in schizophrenia and major depression during acute and chronic disease phases (Hope et al., 2009; Momtazmanesh et al., 2019). Numerous studies have evaluated serum concentrations of cytokines (IL, IFN, TNF), growth transforming factors, and chemokines in BD patients. Research has pointed to an increase in mainly proinflammatory factors i.e., IL-1β, IL-6, and TNF-α (Forrest et al., 2018; Formanova et al., 2019; Kany et al., 2019; Pawluk et al., 2020). On the other hand, several studies have shown that anti-inflammatory cytokines (IL-4, IL-10, IL-13, IGF-1, TGF-β) increase BDNF release and inhibit microglial proinflammatory activity, resulting in increased synaptic pruning and microglial phagocytosis (Barbosa et al., 2011a; Lee et al., 2015; Sochocka et al., 2017; Liu et al., 2019; Milan-Mattos et al., 2019). Recently, some studies have evaluated circulating inflammatory mediators during different phases of BD as potential biomarkers for diagnosis or treatment, and the results were promising (Bhattacharya et al., 2016; Goldsmith et al., 2016; Sigitova et al., 2017; Rowland et al., 2018). Multiple studies have demonstrated an increase in IL-6, IL-1, IL-2, TNF-α, and TNFR1 serums, during the manic and depressive phases, compared to controls and euthymic patients (Brietzke et al., 2009a,b; Barbosa et al., 2011a; Modabbernia et al., 2013; Luo et al., 2016) while IL-4 concentration was significantly lower than in controls (Kim et al., 2007). Nonetheless, other studies have found elevated IL-6 in mania and euthymia, but not in bipolar depression (Uyanik et al., 2015; Jacoby et al., 2016). Kauer-Sant’Anna et al. (2009) found that IL-6 levels were elevated during the advanced stages of disease progression, while Hamdani et al. (2012) reported that the anti-inflammatory IL-10, increased in the early stages of the disease but decreased in the final stages. It is consistent with chronic progressive neuroinflammation, where BD patients with a more significant number of previous episodes, exhibit higher levels of TNF-α and IL-6 during all disease states (Kauer-Sant’Anna et al., 2009).

Other changes in these biomarkers have been observed during different phases following the treatment of acute illness. For instance, levels of the endogenous interleukin receptor antagonist (IL-1RA) were lower in the manic stage among chronic patients, while IL-6 was higher in the euthymic phase but not during the depressive phase compared to controls. Furthermore, IL-1β levels were also significantly elevated in chronic euthymic BD (Hamdani et al., 2013; Goldsmith et al., 2016). This IL-1 activates the transcription factor NF-κB, which in turn enhances the expression and release of IL-6, IL-8, and interferon-gamma (Magalhães et al., 2012; Kany et al., 2019) Thus, Rowland et al. (2018) concluded that while no single biomarker was able to differentiate mood phases or evaluate the stages of the disease, specific combinations including IL-6, BDNF, TNF-α, TNFR1, IL-2, IL-10, and IL-4, were correlated with the disease’s stages. These findings demonstrate a significant link between the immune system and BD pathophysiological pathways.

### TCA Cycle Enzymes and Metabolites

TCA cycle, a crucial component of respiratory metabolism, is composed of a set of eight enzymes present in the mitochondrial matrix. However, most of the TCA cycle enzymes are encoded in the nucleus in higher eukaryotes (Cavalcanti et al., 2014). Studies suggest that mitochondrial dysfunctions play an essential role in the pathophysiology of BD. Increased neuronal oxidative stress produces deleterious effects on signal transduction, plasticity, and cell resilience (Olmez and Ozyurt, 2012), which can induce mitochondrial dysfunctions reducing the ETC activity and of the TCA cycle. The impact of these dysfunctions can be measured in the peripheral blood and postmortem brains of BD patients (De Sousa et al., 2014a; Valvassori et al., 2018). This notion is consistent with parallel transcriptomics, proteomics, and metabolomics studies, showing differential expression of numerous genes related to mitochondrial functions and oxidative stress between patients with mental disorders and healthy controls (Prabakaran et al., 2004). For instance, a loss of function mutation in the malic dehydrogenase enzyme gene, which converts malic acid to pyruvate, was found in the postmortem brains of patients with longer mental disease duration (Lee et al., 2007).

Changes in the TCA cycle, modify brain metabolism, and produce free radicals, leading to further dysfunction. The final product of glycolysis, pyruvate, is converted to acetyl-CoA by pyruvate dehydrogenase. Pyruvate, the end-product of glycolysis, is derived from cellular cytoplasm being destined into mitochondria as fuel undergirding the TCA carbon flux, being critical for mitochondrial ATP generation and for driving several major biosynthetic pathways in TCA (Gray et al., 2014). Acetyl-CoA is then converted to CO_2_ in the TCA cycle with the resulting production of NADH and FADH_2_, the electron donors for the ETC, and ATP production. Eight enzymes control the TCA cycle and inactivating anyone can reduce mitochondrial energy generation (Blass and Brown, 2000; Shi and Tu, 2015; Lazzarino et al., 2019). Despite its importance, few studies have evaluated the activity of the TCA cycle enzymes in patients with mental diseases. Bubber et al. (2011) demonstrated that the activities of enzymes in the TCA cycle varied considerably in the human brain in schizophrenia. They determined, on the prefrontal cortex, the activities of the PDHC, aconitase, isocitrate dehydrogenase (ICDH), and KGDHC. The activity of aconitase was undetectable, and the KGDHC and ICDH activities were very low. On the other hand, fumarase and malate dehydrogenase had the highest activity, while pyruvate dehydrogenase complex (PDHC) and citrate synthase activities were intermediate. Reduced activity of some of these enzymes suggests that some patients with schizophrenia have abnormalities in neural mitochondria. Interestingly, Bubber et al. (2004) had demonstrated that the enzymatic activities of the TCA cycle in mouse brains have a similar pattern, although the majority of the enzyme activities in the brain were 2–3 times higher than in humans brains. However, a study involving 18 untreated BD patients in major depressive episodes found no changes in TCA cycle enzymes compared to controls. The research assayed the activity of the key TCA cycle enzymes citrate synthase, malate dehydrogenase, and succinate dehydrogenase from leukocytes of BD patients (de Sousa et al., 2015). In contrast, Yoshimi et al. (2016) showed that serum levels of pyruvate and α-ketoglutarate in BD patients were significantly higher than those of healthy controls, while serum levels of acetyl-CoA and oxaloacetate were not altered. It is possible that TCA cycle enzymatic alterations are present only during the manic phases. Further, no study has been conducted during later disease stages. Although the BD patients presented higher serum levels of α-ketoglutarate and pyruvate than controls, the reasons underlying are unknown. Increased pyruvate levels likely play a role in the pathogenesis of BD. The α-ketoglutarate is a key metabolite in the TCA, but also an obligatory substrate for 2-oxoglutarate-dependent dioxygenases (2-OGDO) which are involved in DNA and histone methylation, producing an epigenetic impact (Salminen et al., 2014). Altered α-ketoglutarate levels in BD may lead to epigenetic changes. Epigenetic modifications have been suggested to play an important role in the pathogenesis of many psychiatric disorders including BD (Labrie et al., 2012; Kato and Iwamoto, 2014).

Findings from animal model studies also implicate dysregulation of the TCA cycle in mental illness. Valvassori et al. (2014) found reduced levels of the TCA cycle enzyme citrate synthase, succinate dehydrogenase, and malate dehydrogenase in the prefrontal cortex, hippocampus, and striatum in amphetamine-treated rats, a commonly employed animal model of mania. Further, these reduced levels were associated with behavioral hyperactivity.

### Oxidative Stress Markers

The role of oxidative stress in BD pathophysiology has been investigated in several studies. Increased neuronal oxidative stress produces deleterious effects on signal transduction, plasticity, and cell resilience (Olmez and Ozyurt, 2012), which can induce mitochondrial dysfunctions and reduce ETC activity that can be measured in the peripheral blood and postmortem brains of BD patients (De Sousa et al., 2014b; Valvassori et al., 2018). BD is characterized by alterations in CAT, SOD, and GSH-Px activity, NO, and GSH levels, DNA damage, and lipid peroxidation (Gawryluk et al., 2011; Tunçel et al., 2015). Although there is a great deal of work demonstrating serum changes in antioxidant enzymes across all BD stages, these findings are conflicting. Because of these discrepant results, these enzymes cannot yet be used as BD biomarkers, but may nonetheless be useful for evaluating disease stages (Gama et al., 2013).

As related above, studies in animals and humans have reported that increased oxidative damage reduced BDNF expression. However, BDNF has been shown to stimulate the expression and activities of GSH-Px and SOD; resulting in reduced oxidative damage (He and Katusic, 2012; Valvassori et al., 2015; Wang et al., 2020).

Clinical studies in individuals with schizophrenia or BD, provide an empirical basis for hypothesizing that abnormal BDNF and oxidative stress regulation observed in these disorders are inter-related (Fernandes et al., 2011; Zhang et al., 2015; Mansur et al., 2016). However, two recent studies reported that patients with BD exhibited a negative association between serum BDNF levels and lipid peroxidation (Tsai and Huang, 2015; Newton et al., 2017). Also, studies in schizophrenic populations observed a positive correlation between BDNF and TBARS, and a negative association between BDNF and SOD activity (Gama et al., 2008; Zhang et al., 2015). Nonetheless, there is significant heterogeneity in results within and across studies of these systems, which limits the generalizability of the findings. For example, metabolic comorbidities, obesity, and impaired glucose metabolism affect the activities of BDNF and antioxidant enzymes (Tinahones et al., 2009). Studies have demonstrated increased serum SOD activity in patients during manic or depressive episodes (Kunz et al., 2008). Increased SOD activity has been reported in medicated or unmedicated patients during manic episodes (Salim et al., 2011) and acute BD episodes, but not in euthymic patients (Singh et al., 2010). One study found CAT increases in euthymic and manic patients regardless of medication status (Steckert et al., 2010). In contrast, another found that CAT was decreased in euthymic patients but increased in unmedicated manic patients (Raffa et al., 2012). Halliwell (2006) reported increased SOD, CAT, and GSH-Px in patients during manic and depressive BD episodes.

Conversely, Vasconcelos-Moreno et al. (2017) found that GSH-Px activity was reduced in euthymic BD I patients compared to controls. A meta-analysis that included 27 studies, with 971 patients, measured eight peripheral oxidative stress markers in BD. Markers of lipid peroxidation, DNA/RNA damage, and NO were significantly increased in all stages in BD I/II patients compared to healthy controls (Brown et al., 2014; Scola et al., 2016). Andreazza et al. (2010) reported higher levels of mitochondrial protein oxidation in patients with BD, whereas another meta-analysis concluded that TBA-RS levels might be higher during manic or depressive episodes than during remission. Additionally, NO concentrations were elevated in BD patients regardless of mood state (Savas et al., 2006; Siwek et al., 2016).

## Conclusion

Clinical and animal studies have identified multiple promising BD biomarkers that may be related to neuroinflammation, and that may alter its concentrations throughout mood episodes, showing that patients can present increased systemic toxicity during manic and depressive episodes, compared to euthymic patients (Kapczinski et al., 2010, 2011). Despite the systemic toxicity and consequent allostatic overload associated with BD, a causal link between these characteristics has yet to be established. However, the co-occurrence of acute BD episodes, clinical comorbidities, and substance abuse indicates that initial allostatic loading can produce a long-term overload effect. This state of chronic systemic toxicity occurs mainly by the dysregulation of cytokine signaling and the consequent mitochondrial oxidative stress, producing neuroinflammation, which leads to decreased BDNF expression. This observation supports the neuroprogression hypothesis and may explain, at least partially, the deficits associated with chronic BD. However, the mechanisms contributing to lower BDNF are not yet fully understood. It has been suggested that the methylation of BDNF gene promoters, can epigenetically modulate BDNF transcription and that mitochondrial oxidative stress and cytokine levels may alter the binding of nuclear transcription factors (Martinowich et al., 2003). Also, as related above, the brain is particularly susceptible to oxidative damage due to its high rate of oxygen use, lipid constitution, and low antioxidant defenses. In BD, the prevailing hypothesis is that a fundamental disturbance in mitochondrial functions generates a higher oxidative stress load. Changes in Complexes I, II, and III paired with reduced GSH levels have been detected in BD (Andreazza et al., 2010). Further, significant increases in SOD, CAT, and GSH-Px activities have been found, suggesting the induction of compensatory mechanisms to counter the pro-oxidative state. NO levels and oxidative damage to lipids have also been identified as potential systemic toxicity markers in BD patients (Andreazza et al., 2009). These findings support the vital contribution of oxidative stress to BD neuroinflammation, and the clinical neuroprogression, justifying the research on antioxidant mechanisms as new therapeutic strategies (Pandya et al., 2013). Thus, it is hypothesized that the treatment with stabilizers drugs during the early stages of BD, may be beneficial by offering neuroprotection and slowing systemic toxicity progression through the increasing of BDNF. Also, this toxicity may be linked to age and the number of episodes. With each episode, lower BDNF levels result in more significant cognitive impairment and reduced functionality, further reducing the chances of returning to euthymia. Indeed the number of episodes has a more significant impact on disease evolution, than the patient’s age (Passos et al., 2016; Scussel et al., 2016).

Thus, based on our improved understanding of the neuroinflammation and neuroprogression, it is reasonable to speculate that combinations of biomarkers for different pathophysiological processes of BD, will 1 day help predict disease evolution, treatment response, and long-term outcomes (Goldberg and Harrow, 2004). However, the interactions among biomarkers are complex and, as of yet, cannot predict an outcome. Thus, as questioned by Vinberg (2020), in a recent article, the search for peripheral biomarkers in psychiatry is like searching for the needles in a haystack.

Thereby, this review sought to clarify some pathophysiological mechanisms of BD, focusing mainly on the energetic metabolism of brain cells and their correlation with mental diseases.

## Author Contributions

LC and DD: conception of the idea, drafting and revising the article and revising the article and correspondence. LC, OU, and TM: bibliographic research and main drafting. OU: elaboration of figures. All authors contributed to the article and approved the submitted version.

## Conflict of Interest

The authors declare that the research was conducted in the absence of any commercial or financial relationships that could be construed as a potential conflict of interest.
